# Single-cell RNA sequencing unveils tumor heterogeneity and immune microenvironment between subungual and plantar melanoma

**DOI:** 10.1038/s41598-024-57640-8

**Published:** 2024-03-25

**Authors:** Panpan Wang, Yangyang Ma, Yige Zhao, Yong Li, Chenyu Tang, Shiwen Wang, Sha Jin, Jiaqi Wang, Mengyan Zhu, Bo Xie, Ping Wang

**Affiliations:** 1 Fourth Clinical College, Zhejiang Chinese Medical University, Hangzhou, China; 2 Department of Dermatology, Hangzhou Third People’s Hospital, Hangzhou, China; 3 Research Center, Shanghai Yeslab Biotechnology, Shanghai, China

**Keywords:** Cancer genomics, Melanoma

## Abstract

Acral melanoma (AM) is a subtype of melanoma with high prevalence in East Asians. AM is characterized by greater aggressiveness and lower survival rates. However, there are still fewer studies on immune mechanisms of AM especially subungual melanoma (SM) versus non-subungual melanoma (NSM). In order to explore tumor heterogeneity and immune microenvironment in different subtypes of AM, we applied single-cell RNA sequencing to 24,789 single cells isolated from the SM and plantar melanoma (PM) patients. Aspects of tumor heterogeneity, melanocytes from PM and SM had significant differences in gene expression, CNV and pathways in which tumor-associated such as NF-kb and Wnt were involved. Regarding the immune microenvironment, PM contained more fibroblasts and T/NK cells. The EPHA3-EFNA1 axis was expressed only in cancer-associated fibroblast (CAF) and melanocytes of PM, and the TIGIT-NECTIN2 axis was expressed in both AM subtypes of T/NK cells and melanocytes. Altogether, our study helps to elucidate the tumor heterogeneity in AM subpopulations and provides potential therapeutic targets for clinical research.

## Introduction

Acral melanoma (AM) is a subtype of melanoma that occurs in the skin of non-exposed areas such as palm, sole, and subungual areas. AM has unique characteristics which are differently from non-acral melanoma (NAM) in genotype, clinical presentation, treatment and prognosis^1^. BRAF and NRAS gene mutations are common in NAM; however, only 15.5% of AM carries a BRAF gene mutation, and therefore the majority of AM patients do not benefit from targeted therapies^2^. AM has a higher probability of being mutated in KIT and CDK 4/6, and a recent study showed that 20% of patients in a phase II clinical trial of Palbociclib for AM with CDK pathway mutations had tumor shrinkage^3^, another phase II clinical trial shows Imatinib ineffective in KIT-mutated AM^4^. Anti-PD-1 antibodies are a commonly used in advanced melanoma, and it reported that the efficacy of immunotherapy was limited poorer response and survival in patients with subungual melanoma^5^. Interferon-α (IFN-α) upregulates PD-L1 expression and enhances the antitumor effects of CD8+ T cells though the tumor microenvironment^6^. IFN-α combined with PD-1 inhibitors may be feasible for the treatment of advanced AM, but effective treatments for AM are still lacking.

Currently, there are more studies on SM subtypes. In an AM Whole-genome sequencing, SM lacked BRAF and PTEN mutations and was more likely to have SPRED 1 rearrangements or CDK 4 aberrations^7^. In a Clinical and molecular features analysis of 54 SM and 78 NAM cases, KIT, KRAS mutations, and CDK4/CCNDl amplification were predominantly seen in SM, whereas BRAF, NRAS mutations and CDKN 2A/B loss were seen almost exclusively in NAM. In the PI3 K/mTOR pathway, RICTOR amplification and TSC1K587 R mutation were only in SM, PTEN loss and AKT mutation were only in NAM^8^. The molecular and cell clusters mechanism in AM is not lucid, and especially the difference between the AM especially subungual melanoma (SM) versus non-subungual melanoma (NSM) should be further studied.

In this study, we compared stage IIC plantar melanoma (PM) and SM by single-cell sequencing and other methods to further explore the intrinsic characteristics of different subtypes of AM in terms of tumor heterogeneity and immune environment. Based on the evidence of copy number variations (CNVs), we identified the subpopulations of malignant melanocytes. We compared differentially expressed genes (DEGs), CNVs and gene set variation analysis (GSVA) of PM and SM malignant melanocytes with significant differences. We analyzed cell chat between tumor cells and other cells, the results show that the EPHA3-EFNA1 axis was enriched between CAF-NPY1R and tumor cells, mainly expressed in PM1. The TIGIT-NECTIN2 axis is highly expressed in both PM and SM, suggesting a possible role in the development of AM. In conclusion, our study demonstrates tumor heterogeneity between PM and SM, redefines the T/NK and tumor cell roadmap in the AM ecosystem, and provides new potential strategies for targeting AM TME.

## Materials and methods

### Sample collection

Two acral melanoma patients were recruited for this study, including PM and SM cases. Both samples were obtained from Hangzhou Third Hospital Affiliated to Zhejiang Chinese Medical University, Hangzhou, China. Both patients were stage IIC T4bN0M0, SM patient was a 70 years old female and PM patient was a 68 years old male. One tumor tissue and one adjacent normal tissue were collected from each patient.

### Ethics approval

All experimental procedures were approved by the Ethics Committee of Hangzhou Third Hospital Affiliated to Zhejiang Chinese Medical University and were conducted in compliance with the Helsinki Declaration, under protocol number 2023KA065. Informed consents were obtained from both two patients.

### Single-cell RNA sequencing (scRNA-seq)

Two tumor tissues and two adjacent normal tissues derived from two patients were used for scRNA-seq. All four fresh tissue samples were collected and immediately stored in the GEXSCOPE Tissue Preservation Solution (Singleron Biotechnologies) at 2–8 °C. Before tissue dissociation, the specimens were washed with Hanks Balanced Salt Solution three times and minced into 1–2 mm pieces. More detailed procedure on scRNA-seq were descripted in “Supplemental Materials and Methods”.

### Immunofluorescence staining

Immunofluorescence staining was conducted to detect the expression of proteins and examine the subcellular localization of EFNA1, CD226, NECTIN2, and TIGIT. Tissue biopsies of melanoma tumor tissues were deparaffinized and rehydrated, followed by antigen retrieval. After 30 min blocking in 3% bovine serum albumin (BSA), tissues were incubated overnight at 4 °C with the following primary antibodies: rabbit anti-EFNA1 antibody (1:200, Cusabio Cat# CSB-PA002367), rabbit anti-CD226 antibody (1:200, Cusabio Cat# CSB-PA006679), rabbit anti-TIGIT antibody (1:100, Cusabio Cat# CSB-PA675446LA01HU), and rabbit anti-NECTIN2 antibody (1:200, Proteintech Cat No. 27171–1-AP). The secondary antibodies subsequently were added for 50 min followed by counterstaining with DAPI. Tissues were then observed and photographed under the inverted microscope.

## Results

### Single-cell analysis revealed the complexity of AM

**Figure 1:**
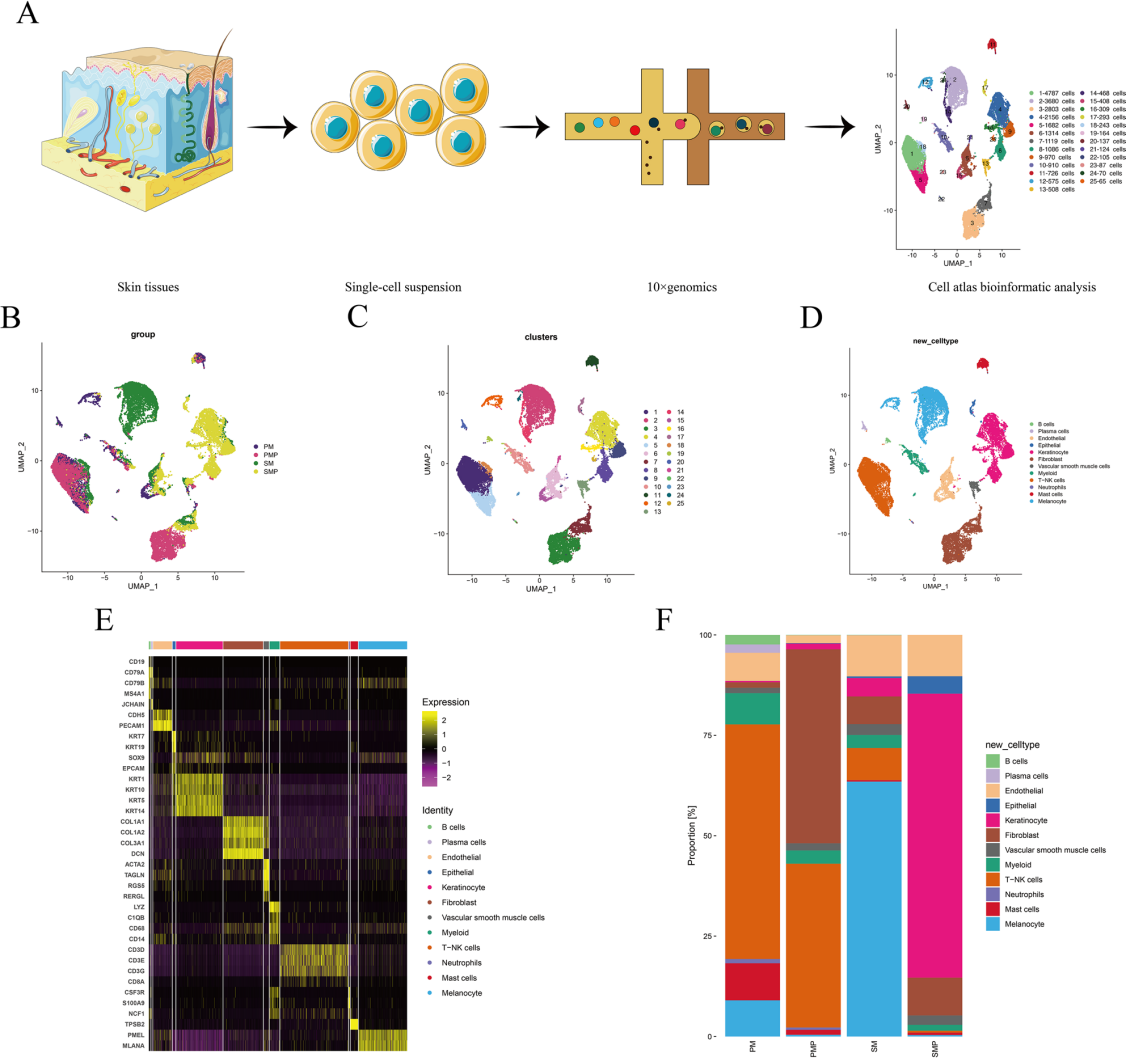
Comprehensive cellular overview of AM. (**A**) Schematic diagram of scRNA-seq analysis workflow. AM and adjacent tissues were dissociated into single cells, sequenced using 10 × Genomics platform. (**B)** UMAP plots for the 4 tissues. (**C**) UMAP plots for the 25 clusters. (**D**) UMAP plots for the 12 cell types. (**E**) Heatmap of functional gene sets in 12 cell types. (**F**) Average proportion of each cell type between 4 tissues.

In order to explore the tumor heterogeneity and immune environment at the single-cell level, we collected four tissues from two AM patients with clinical stage IIC T4bN0M0, including SM patient, SM adjacent normal tissues (SMP), PM patient and PM adjacent normal tissues (PMP) (Fig. 1A,B). For data analysis, PM1 stands for the merging of PM and PMP, and SM1 stands for the merging of SM and SMP. As a result, 24,789 individual cells from four samples were passed quality control criteria (Supplementary Table 1) and were divided into 25 major clusters (Fig. 1C). Twelve clusters were explored with unbiased clustering across all cells by Principal Components Analysis (PCA) and visualized by Uniform Manifold Approximation and Projection (UMAP) (Fig. 1D). We annotated the cell type of each cluster with the well-established gene markers, including B cells, plasma cells, endothelials, epithelials, keratinocytes, fibroblasts, vascular smooth muscle cells, myeloid cells, T/NK cells, neutrophils, mast cells and melanocytes (Fig. 1E). The proportion of the 12 cell populations varied among the samples, with more melanocytes and fewer T/NK cells in SM than PM, more keratinocytes and fewer fibroblasts in SMP than PMP; however, cell types such as B-cells and plasma cells were rare in all four samples (Fig. 1F and Supplementary Table 2).

### Single-cell analysis revealed the tumor heterogeneity of melanocytes

**Figure 2:**
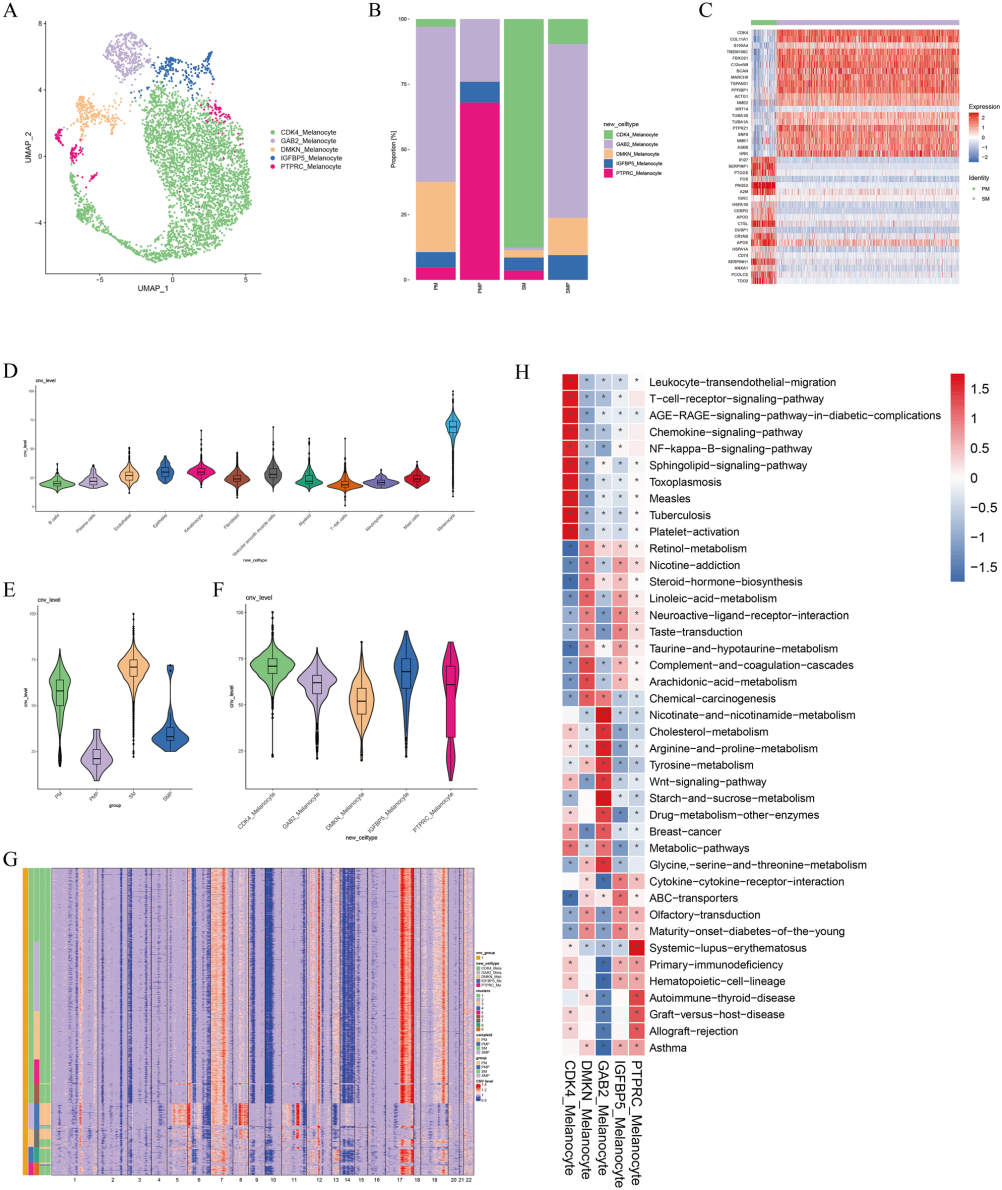
Identification and characterization of malignant cells in AM. (**A**) UMAP plots for the 5 subpopulations of melanocytes. (**B**) Average proportion of each subpopulation of melanocytes between 4 tissues. (**C**) Heatmap showing the top 20 DEGs (Wilcoxon test) between PM and SM. (D) Violin plots showing the CNV signals for the 12 cell types. (**E**) Violin plots showing the CNV signals for the 4 tissues. (**F**) Violin plots showing the CNV signals for the 5 malignant cell subpopulations. (**G**) Heatmap showing CNVs for individual cells of melanocytes. The color shows the log2 CNV ratio. Red: amplifications; blue: deletions. (**H**) Differences in pathway activity (scored per cell by GSVA) in 5 malignant cell subpopulations.

We clustered the melanocyte cells and revealed 5 distinct subpopulations by visualization using UMAP. The cell types were defined based on the specifically expressed genes in each cluster, including CDK4, GAB2, DMKN, IGFBP5 and PTPRC (Fig. 2A). Melanocytes were predominantly found in two tumor tissues, where CDK4 subpopulation was predominantly expressed in SM and GAB2 subpopulation was predominantly expressed in PM (Fig. 2B). It is showed that the distribution of cell clusters from SM and PM were in a high degree of tumor heterogeneity. Analysis of DEGs showed that PM highly expressed SERPINF1, and SM highly expressed CDK4, COL11A1, and S100A4. Although PM and SM expressed different genes, all of these genes were epithelial mesenchymal transition (EMT) characteristic (Fig. 2C).

Malignant cells were identified by inferring large-scale CNVs with immune and stromal cells as references (Fig. 2D). We found that SM has higher CNV signal than PM (Fig. 2E). Among the five subpopulations of melanocytes, the CDK4 subpopulation had the highest CNV signal, indicating the highest degree of malignancy (Fig. 2F). Amplification of chromosome 7, 12, 17 and deletion of chromosome 6, 9, 10, 14 were detected in SM. Amplification of chromosome 6, 8, 11 and deletion of chromosome 13 were detected in PM (Fig. 2G). Interestingly, we found that the CDK4 is located on chromosome 12, and both GAB2 and PAK1 are located on chromosome 11. Similarly, the highly expressed pathways in GSVA in SM are quite different from the PM. CDK4 subpopulation is primarily located in SM, highly expressing the NF-kb pathway, whereas the GAB2 subpopulation, which is primarily located in PM, predominantly expresses the Wnt pathway (Fig. 2H).

### CAFs were enriched in the AM tumors

**Figure 3:**
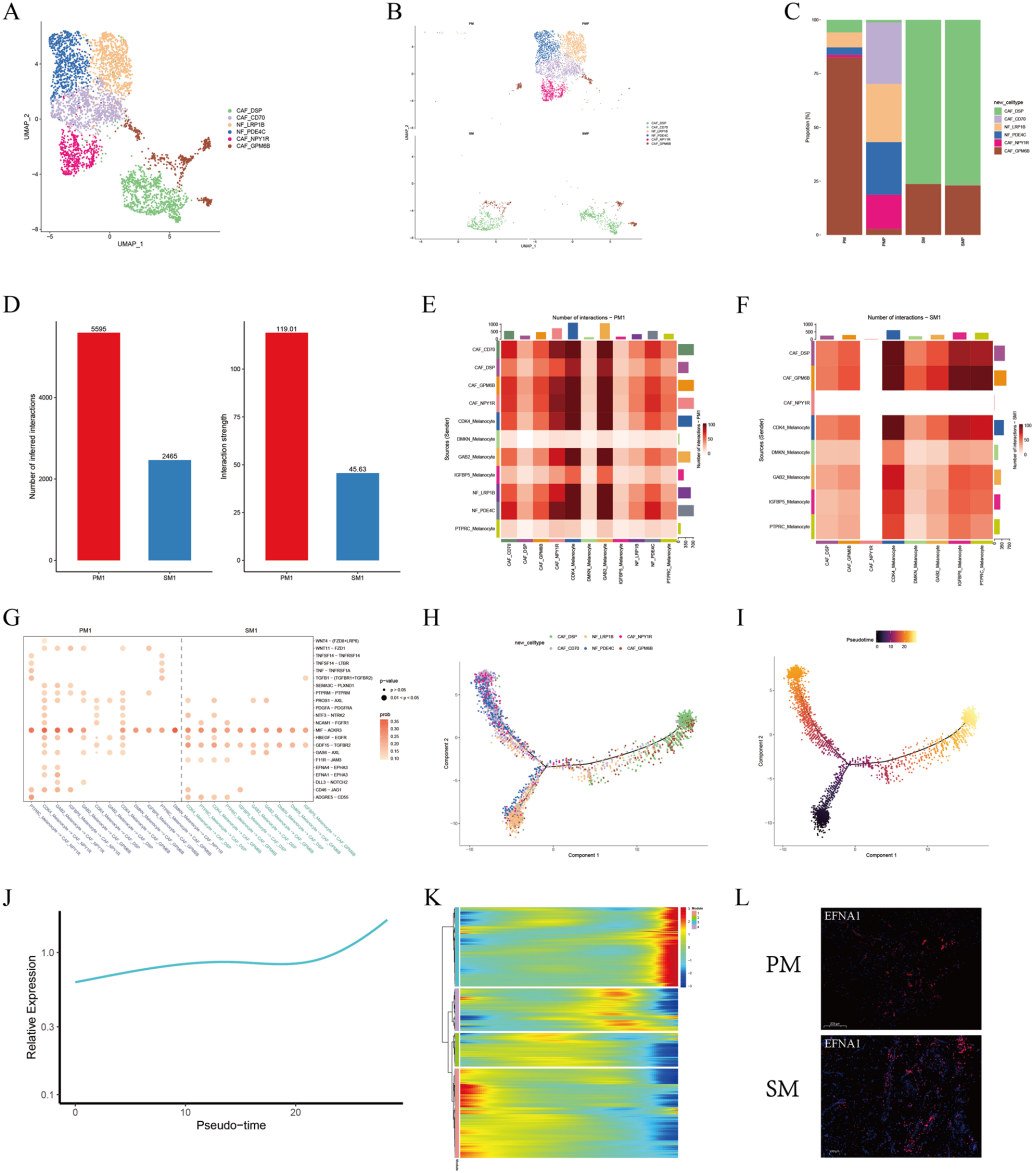
Detailed characterization of fibroblasts. (**A**) UMAP plots for the 6 subpopulations of fibroblasts. (**B**,**C**) Average proportion of each subpopulation of fibroblasts between 4 tissues. (**D**) Bar plot showing the number and strength of interactions between melanocytes and fibroblasts in PM1 and SM1. (**E**,**F**) Heatmap showing the number of interactions between melanocytes subpopulations and fibroblasts subpopulations in PM1 and SM1. (**G**) Dot plot showing receptor-ligand pair analysis of the interactions between melanocytes and fibroblasts. (**H**,**I**) Pseudotime trajectory demonstrating the transcriptome lineage of 6 fibroblasts subpopulations. Colors indicate pseudotime progression. (**J**) Line plot showing the development trend of module 3 in pseudotime trajectory. (**K**) The development trend of 4 modules of fibroblasts in pseudotime trajectory. (**L**) Immunofluorescence staining of EFNA1 in PM and SM.

The tumor microenvironment (TME) is a complex ecosystem composed of various cell types including malignant and stromal cells^9^. Fibroblasts are common cells of the connective tissue and can be activated in cancer development, which are commonly known as cancer-associated fibroblasts (CAFs). CAFs play an important role in TEM that can interact with cancer cells to promote tumor metastasis and progression^10^. The CAFs gene marker fibroblast activation protein (FAP) was used to distinguish the normal fibroblasts (NFs) and CAFs, and six subpopulations NF-LRP1B, NF-PDE4C, CAF-DSP, CAF-CD70, CAF-NPY1R and CAF-GPM6B were identified (Fig. 3A). Most of the fibroblasts subpopulations are present in the PMP including CAF-CD70, CAF-NPY1R and NFs (Fig. 3B,C).

We performed cellchat analysis to explore the interaction between CAFs and malignant cells. The results showed that the number and strength of fibroblast-melanocytes interactions were higher in PM1, in which the CAF-NPY1R subpopulation was the most abundant (Fig. 3D,F). We found that EPHA3 is expressed only in CAF-NPY1R subpopulation. The ligand EFNA1 is higher expressed than EFNA4 in malignant cells (Fig. 3G and Supplementary Table 3).

For exploring the evolution of EPHA3 in six fibroblast subpopulations, we performed pseudo-time analysis. It showed that above six subpopulations were in different developmental states. As a starting point of NFs, CAFs in PM1 and SM1 differentiate in two directions, subpopulation CAF-NPY1R/CD70 and CAF-DSP were mainly at the two roots of phylogenetic tree (Fig. 3H). The color of CAF-DSP subpopulation which is predominantly present in SM1 is lighter, indicating later differentiation (Fig. 3I). Summarize genes to form 4 modules based on differences in temporal developmental trajectories, and EPHA3 was located in module 3 (Supplementary Table 4). The significance of expression is that EPHA3 expression is elevated with the evolution of fibroblasts (Fig. 3J,K). We verified that the ligand EFNA1 was highly expressed in AM using immunofluorescence staining (Fig. 3L). It suggests that blocking the EPHA3-EFNA1 axis may affect the interaction of CAF with malignant cells and could be an effective therapeutic strategy for PM.

### Distinct T/NK subpopulations in AM ecosystem

**Figure 4:**
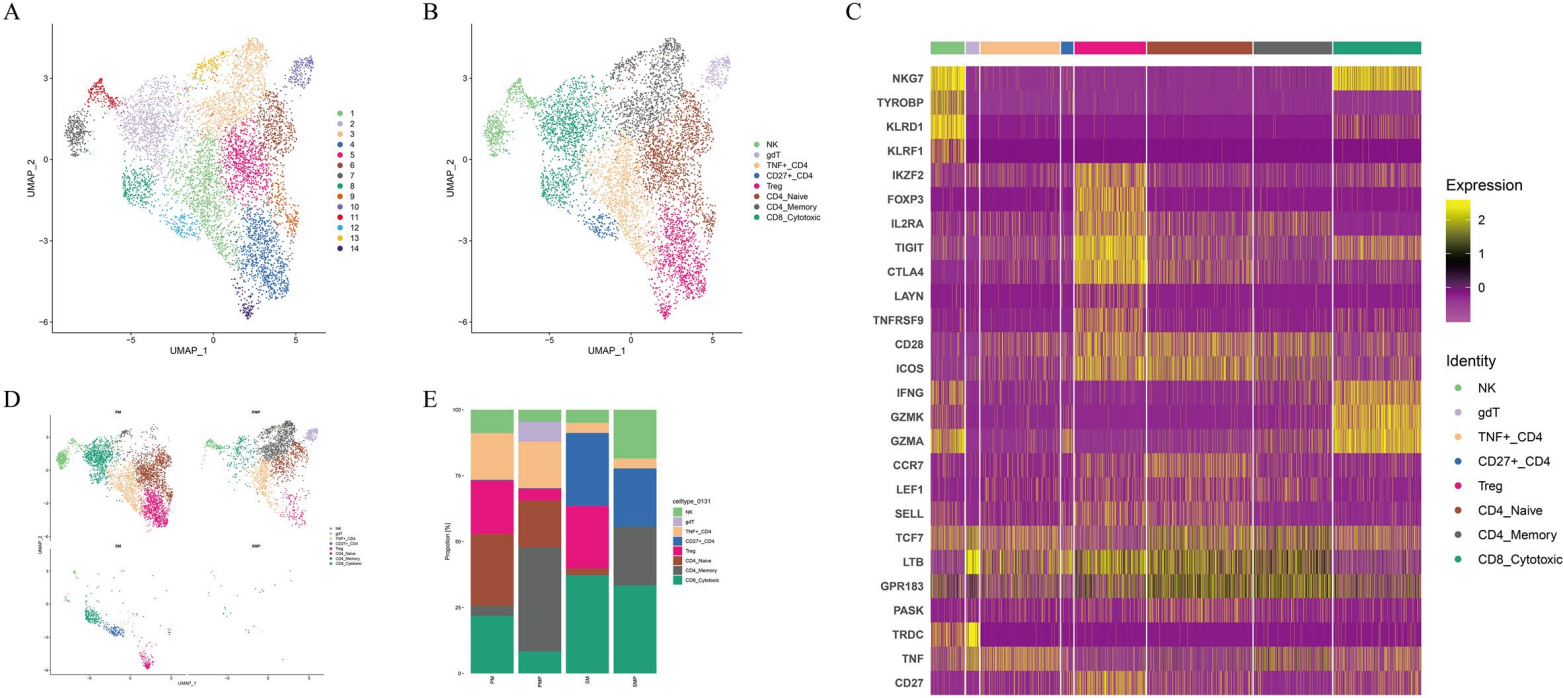
Distinct T/NK cells subpopulations distribution in AM. (**A**) UMAP plots for the 14 clusters of T/NK cells. (**B**) UMAP plots for the 8 cell types of T/NK cells. (**C**) Heatmap of functional gene sets in 8 cell types of T/NK cells. (**D**,**E**) Average proportion of each subpopulation of T/NK cells between 4 tissues.

In tumor immunity, tumor cells act as antigens while immune cells and leukocytes infiltrates the tumor tissue function through chemotaxis for immune defense^11^. Immune escape also is an important factor in tumorigenesis. According to gene expression, T/NK cells are divided into 8 subpopulations, namely NK, gdT, Treg, CD8+ Cytotoxic, TNF+ CD4+ , CD27+ CD4+ , CD4+ Naive and CD4+ Memory (Fig. 4A,B). IL2RA, FOXP3, IKZF2, co-stimulatory (CD28, TNFRSF9 and ICOS) and inhibitory markers (TIGIT, CTLA4 and LAYN) were highly expressed in the Treg subpopulation. CD4-Naïve T cells were marked with expression of CCR7, LEF1, SELL and TCF7 genes. CD4-Memory T cells were featured with high expression of LTB, GPR183 and PASK. CD8-Cytotoxic were characterized with high expression of GZMK, GZMA, IFNG and NKG7. The NK subpopulation was marked with high expression of NKG7, TYROBP, KLRD1 and KLRF1 (Fig. 4C). We found that SM had a stronger immunosuppressive status compared with PM. Unlike fibroblasts, tumor tissues had more T cells infiltration than adjacent normal tissues (Fig. 4D,E). SM1 lacked NK cells, which may be related to the poor prognosis of SM patients.

### The TIGIT-NECTIN2 axis was enriched in the interplay between T/NK cells and melanocytes

**Figure 5:**
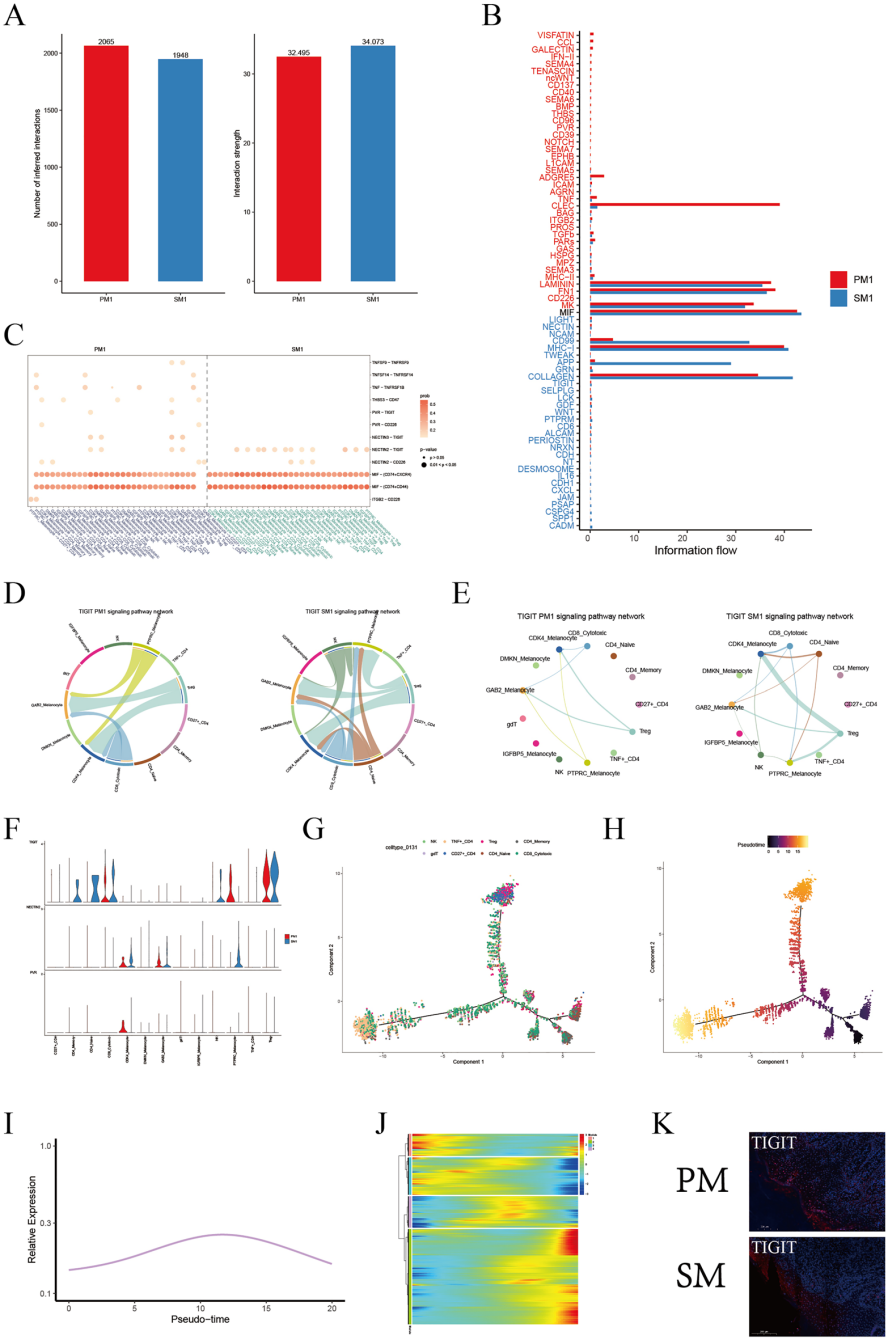
T/NK cells interact with melanocytes via the TIGIT-NECTIN2 axis. (**A**) Bar plot showing the number and strength of interactions between melanocytes and T/NK cells in PM1 and SM1. (**B**) Differences in interactions in the information flow of PM1 and SM1. (**C**) Dot plot showing receptor-ligand pair analysis of the interactions between melanocytes and T/NK cells. (**D**,**E**) Cell chat showing the interactions between TIGIT and its ligand in PM1 and SM1. The thickness of the lines represents strength. (**F**) Violin plots showing the expression intensity of TIGIT and its ligand NECTIN2/PVR in melanocytes subpopulations and T/NK cells subpopulations. (**G**,**H**) Pseudotime trajectory demonstrating the transcriptome lineage of 8T/NK cells subpopulations. Colors indicate pseudotime progression. (**I**) Line plot showing the development trend of module 4 in pseudotime trajectory. (**J**) The development trend of 4 modules of T/NK cells in pseudotime trajectory. (**K**) Immunofluorescence staining of TIGIT in PM and SM.

To explore the interactions between melanocytes and T/NK cells, we conducted cellchat analyses based on ligand-receptor pairs. In cellchat, the number of melanocytes and T/NK cells cellular interactions was greater in PM1, whereas the strength of the effect of SM1 was greater (Fig. 5A). As seen in the information flow, LAMININ, FN1, MK, MIF, MHC-1 and COLLAGEN pathways were highly expressed both in PM1 and SM1. ADGRE5 and CLEC pathways were significantly increased in PM1. CD99 and APP pathways were more expressed in SM1. The differently expressed pathways could provide clues for the therapeutic strategies in PM and SM (Fig. 5B). In addition, the TIGIT/NECTIN2/NECTIN3/PVR axis was enriched in the interactions between T/NK cells and melanocytes, which was a direct cell–cell interaction (Fig. 5C and Supplementary Table 5). TIGIT was predominantly expressed in Treg and CD8-Cytotoxic of PM1, and in NK, Treg and CD8-Cytotoxic of SM1 (Fig. 5D,E). Here we found that TIGIT expression was significantly higher on CD8+ T cells in PM subpopulations than on those in PMP subpopulations. While TIGIT expression in NK cells in PM subpopulations was not significantly different from PMP subpopulations. TIGIT ligands have NECTIN2, NECTIN3, and PVR in PM1 and only NECTIN2 in SM1, in which the TIGIT/NECTIN2 pair is expressed more strongly, which differs from previous TIGIT/PVR expression (Fig. 5F).

In T/NK pseudo-time analysis, naive was used as the starting point to make the proposed time trajectory plot, with darker to lighter colors indicate earlier and later differentiation. It was showed that TNF+ CD4+ and CD27+ CD4+ were located at the terminal ends of the 2 branches (Fig. 5G,H). Pooling T cell expressed genes according to differences in temporal developmental trajectories, TIGIT is located in module 4 (Fig. 5I,J and Supplementary Table 6). TIGIT expression was elevated and then decreased in the proposed temporal differentiation, consistent with the aforementioned enrichment in Treg, NK and CD8-Cytotoxic, which is not expressed in CD4+ T in our results. The expression of TIGIT in AM was validated by immunofluorescence staining (Fig. 5K).

### Downregulation of CD226 in Treg and CD8+ Cytotoxic cells

**Figure 6:**
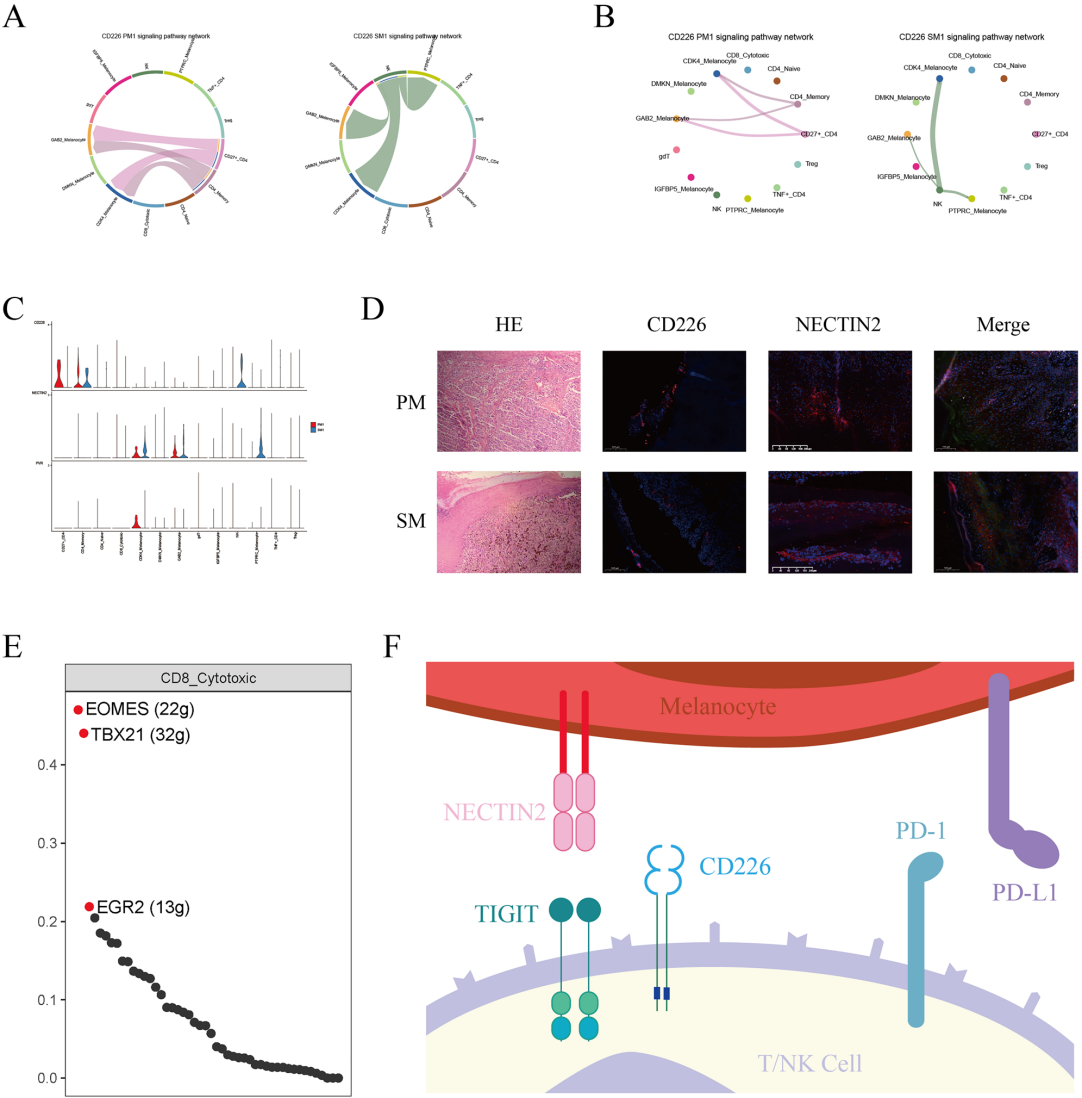
The expression of CD226-NECTIN2 axis in melanocytes and T/NK cells. (**A**,**B**) Cell chat showing the interactions between CD226 and its ligand in PM1 and SM1. The thickness of the lines represents strength. (**C**) Violin plots showing the expression intensity of CD226 and its ligand NECTIN2/PVR in melanocytes subpopulations and T/NK cells subpopulations. (**D**) Hematoxylin–eosin (HE) staining of PM and SM (× 100). Immunofluorescence staining of CD226 and NECTIN2 in PM and SM. Triple immunofluorescence staining of TIGIT, CD226, and NECTIN2. (**E**) SCENIC showing the main TFs of CD8+ Cytotoxic. (**F**) The interaction of TIGIT family receptors and ligands.

CD226 is an inhibitory receptor and competes the same ligands with TIGIT^12^. In the interactions between melanocytes and T-NK cells, CD226 is differentially expressed in each subpopulations in AM. In PM1, CD226 was expressed in CD27+ CD4+ and CD4+ Memory subpopulations, and corresponding ligands in tumor cells were TIGB2, NECTIN2 and PVR. NECTIN2 was expressed in a highest intensity. In SM1, CD226 was expressed in NK cells, and the corresponding ligand in tumor cells was only NECTIN2 (Fig. 6A–C). NECTIN2 were highly expressed in AM, but CD226 was down regulated by immunofluorescence staining (Fig. 6D). We found that CD226 was not expressed in Treg and CD8+ Cytotoxic cells both in PM1 and SM1, and it has reported that the activation receptor CD226 is a key component of T cell biology, and its absence impairs the responsiveness of CD8+ T cells to TCR stimulation^13^.

Beyond that, Eomes is a transcription factor (TF) with a key role in CD8+ T cell differentiation, by binding to the promoter of TIGIT, upregulating its expression. Overexpression of Eomes and losing of CD226 are related. In our SCENIC results, Eomes was enriched in CD8-Cytotoxic (Fig. 6E). In conclusion, CD226 was barely expressed in Treg and CD8+ Cytotoxic cells, while TIGIT was highly expressed in Treg , CD8+ Cytotoxic cells and NK cells (SM1), suggesting that the TIGIT-NECTIN2 axis plays an important role in the immune environment of AM (Fig. 6F).

## Discussion

In this study, we employed scRNA-seq platform to investigate the transcriptomic landscape of different subtypes of AM and revealed novel cellular interactions between tumor cells and T/NK cells. AM was often compared with NAM to find potential therapeutic targets for AM in previous study using single-cell sequencing. Differences in immune response in different subtypes of AM (especially subnail and plantar) in immunotherapy^5^. In order to reveal the tumor heterogeneity and immune environment between SM and PM, we performed a higher level of comparative analysis by cellchat, SCENIC and pseudo-time analysis for understanding their difference traits in cell evolution, transcription factors, and cellular interactions with the microenvironment.

In the AM mutant landscape, IFI27 and SERPINF1 were highly expressed in PM, CDK4, COL11A1 and S100A4 were highly expressed in SM. Among them, CDK4 was most highly expressed in SM, while it was not significantly expressed in PM, which suggests that CDK 4/6 inhibitors may have better SM efficacy of CDK 4/6 inhibitors in SM. Cyclin-dependent kinase 4 (CDK4) pathway is a frequently altered signaling in many cancer types^14^. A study of 514 acral melanomas revealed that alterations in the CDK4 pathway were frequent and that these alterations promoted G1 to S cell cycle transition and tumor progression^15^. Recent studies have shown that combination treatment of CDK4/6 plus MEK inhibitors as first- and second-line therapy in patients with advanced AM^16^. Besides, we found that SM is higher CNV signal and lower T/NK cells, which suggests that SM may have strong characteristics of invasiveness and metastasis and have a worse prognosis, in agreement with the study of Holman BN et al.^8^.

CAF is critical for melanoma initiation and progression^17^. We observed that the EPHA3-EFNA1 pair was enriched between CAF-NPY1R and malignant cells. Eph receptors make up the largest family of receptor tyrosine kinases (RTKs) and bind to ligands in a cell–cell manner^18^. Mary E. Vail et al. showed that EPHA3 is expressed in multiple CAFs subpopulations and promotes tumor growth and angiogenesis^19^. EFNA1 widely participates in tumorigenesis by influencing tumor angiogenesis, malignant cell events and invasiveness^20^. Its expression is upregulated in many kinds of cancers (e.g. gastric cancer^21^, colorectal cancer^22^ and renal cancer^23^), and it is closely related to the prognosis of many tumors^24^. It implies that blocking the EPHA3-EFNA1 pair may benefit the therapy of PM.

Tyrosine-based inhibition motif domain (TIGIT) is an important immune checkpoint for T/NK cells^25^. It has been found that TIGIT can be detected at breast cancer^26^, renal cell carcinoma^27^, lung adenocarcinoma^28^, hepatocellular carcinoma^29^, gastric cancer^30^, acute myeloid leukemia^31^, melanoma^32^ and promote tumor onset, progression and prognosis^33,34^. TIGIT is expressed on human tumor-infiltrating CD8+ T cells, NK cells, Th and Treg cells in melanoma^35^. Our results showed that TIGIT was highly expressed both in PM1 and SM1, mainly in NK, Treg and CD8-Cytotoxic cells. TIGIT acts directly on T cells and inhibits CD8+ T cell proliferation and activation by attenuating T cell receptor (TCR)-driven activation signals^36^. In NK cells, TIGIT binds to the ligand PVR and downregulates NK cell activity ^37^. TIGIT is highly expressed in mouse and human Treg cells^38^, TIGIT+Treg is more inhibitory than TIGIT-Treg in melanoma patients^39^. In addition to this, TIGIT promotes TIGIT immunosuppression by competing with CD 226 for PVR ligands^40^. Binding of CD 226 to PVR leads to phosphorylation of FOXO 1, which promotes the activity of NK cells, CD8+ T cells and Treg cells^41^. Animal experiments showing that TIGIT has only one ligand, PVR, and that tumors are better killed in vitro and in vivo in the absence of TIGIT^42^. TIGIT is used as a targeted immune checkpoint in AM in the latest AM single-cell sequencing^43^. The main ligands of TIGIT are PVR, NECTIN2, NECTIN3 and NECTIN4. In contrast to the results of the melanoma studies described above, the primary ligand for TIGIT and CD226 on tumor cells in our results was NECTIN2. In a single-cell sequencing of breast cancer, NECTIN2-TIGIT mediated interactions between metastatic breast cancer cells and TME cells that promote immune escape and lymph node metastasis^26^. The role of the NECTIN2-TIGIT axis in melanoma of the extremities still needs further validation.

Taken together, our results uncover the tumor heterogeneity and immune environment of different subtypes of AM SM versus PM. Our findings may be a valuable resource to contribute to a deeper understanding of AM-related mechanisms. The factors involved in the results are potential biomarkers for the diagnosis and prognosis, and it may benefit the therapy of AM patients with more potential therapeutic targets. However, our sample size was limited (only two patients), more cases and samples and replicated studies are needed to confirm our conclusions.

### Supplementary Information

Supplementary Legends.

Supplementary Information.

Supplementary Table 1.

Supplementary Table 2.

Supplementary Table 3.

Supplementary Table 4.

Supplementary Table 5.

Supplementary Table 6.

## Data Availability

The raw sequence data reported in this paper have been deposited in the Genome Sequence Archive (Genomics, Proteomics & Bioinformatics 2021) in National Genomics Data Center (Nucleic Acids Res 2022), China National Center for Bioinformation / Beijing Institute of Genomics, Chinese Academy of Sciences (GSA-Human: HRA006100) that are publicly accessible at https://ngdc.cncb.ac.cn/gsa.

## Abbreviations

AM: Acral melanoma
NAM: Non-acral melanoma
SM: Subungual melanoma
PM: Plantar melanoma
CNV: Copy number variation
DEG: Differentially expressed gene
GSVA: Gene set variation analysis
IFN-α: Interferon-α
EMT: Epithelial mesenchymal transition
scRNA-seq: Single-cell RNA sequencing
TME: Tumor microenvironment
Treg: Regulatory T cell
CAF: Cancer-associated fibroblast

## References

[1] Augustin RC (2023). Identification of tumor-intrinsic drivers of immune exclusion in acral melanoma. J. Immunother. Cancer.

[2] Si L (2012). Prevalence of BRAF V600E mutation in Chinese melanoma patients: Large scale analysis of BRAF and NRAS mutations in a 432-case cohort. Eur. J. Cancer.

[3] Mao L (2021). Palbociclib in advanced acral melanoma with genetic aberrations in the cyclin-dependent kinase 4 pathway. Eur. J. Cancer.

[4] Hodi FS (2013). Imatinib for melanomas harboring mutationally activated or amplified KIT arising on mucosal, acral, and chronically sun-damaged skin. J. Clin. Oncol..

[5] Nakamura Y (2020). Anti-PD1 checkpoint inhibitor therapy in acral melanoma: A multicenter study of 193 Japanese patients. Ann. Oncol..

[6] Guo J (2019). Empowering therapeutic antibodies with IFN-α for cancer immunotherapy. PLoS ONE.

[7] Newell F (2020). Whole-genome sequencing of acral melanoma reveals genomic complexity and diversity. Nat. Commun..

[8] Holman BN (2020). Clinical and molecular features of subungual melanomas are site-specific and distinct from acral melanomas. Melanoma Res..

[9] Whiteside TL (2008). The tumor microenvironment and its role in promoting tumor growth. Oncogene.

[10] Schoepp M, Ströse AJ, Haier J (2017). Dysregulation of miRNA expression in cancer associated fibroblasts (CAFs) and its consequences on the tumor microenvironment. Cancers (Basel).

[11] Angell H, Galon J (2013). From the immune contexture to the Immunoscore: The role of prognostic and predictive immune markers in cancer. Curr. Opin. Immunol..

[12] Worboys JD (2023). TIGIT can inhibit T cell activation via ligation-induced nanoclusters, independent of CD226 co-stimulation. Nat. Commun..

[13] Weulersse M (2020). Eomes-dependent loss of the co-activating receptor CD226 restrains CD8+ T cell anti-tumor functions and limits the efficacy of cancer immunotherapy. Immunity.

[14] Sheppard KE, McArthur GA (2013). The cell-cycle regulator CDK4: An emerging therapeutic target in melanoma. Clin. Cancer Res..

[15] Kong Y (2017). Frequent genetic aberrations in the CDK4 Pathway in acral melanoma indicate the potential for CDK4/6 inhibitors in targeted therapy. Clin. Cancer Res..

[16] Rebecca V (2023). ERK hyperactivation serves as a unified mechanism of escape in intrinsic and acquired CDK4/6 inhibitor resistance in acral lentiginous melanoma. Res. Sq..

[17] Van Hove L, Hoste E (2022). Activation of fibroblasts in skin cancer. J. Invest. Dermatol..

[18] Talia M (2023). The Ephrin tyrosine kinase a3 (EphA3) is a novel mediator of RAGE-prompted motility of breast cancer cells. J. Exp. Clin. Cancer Res..

[19] Vail ME (2023). Inhibition of EphA3 expression in tumour stromal cells suppresses tumour growth and progression. Cancers (Basel).

[20] Miao H (2015). EphA2 promotes infiltrative invasion of glioma stem cells in vivo through cross-talk with Akt and regulates stem cell properties. Oncogene.

[21] Nakamura R (2005). EPHA2/EFNA1 expression in human gastric cancer. Cancer Sci..

[22] Shi Z-Z (2012). Genomic profiling of rectal adenoma and carcinoma by array-based comparative genomic hybridization. BMC Med. Genom..

[23] Toma MI (2014). Lack of ephrin receptor A1 is a favorable independent prognostic factor in clear cell renal cell carcinoma. PLoS ONE.

[24] Miyazaki K (2013). EphA4 is a prognostic factor in gastric cancer. BMC Clin. Pathol..

[25] Tang W, Chen J, Ji T, Cong X (2023). TIGIT, a novel immune checkpoint therapy for melanoma. Cell Death Dis..

[26] Xu K (2021). Single-cell RNA sequencing reveals cell heterogeneity and transcriptome profile of breast cancer lymph node metastasis. Oncogenesis.

[27] Takamatsu K (2021). Profiling the inhibitory receptors LAG-3, TIM-3, and TIGIT in renal cell carcinoma reveals malignancy. Nat. Commun.

[28] Sun Y (2020). Combined evaluation of the expression status of CD155 and TIGIT plays an important role in the prognosis of LUAD (lung adenocarcinoma). Int. Immunopharmacol..

[29] Liu X (2019). PD-1+ TIGIT+ CD8+ T cells are associated with pathogenesis and progression of patients with hepatitis B virus-related hepatocellular carcinoma. Cancer Immunol. Immunother..

[30] Ma J (2022). Bioinformatics-guided analysis uncovers TIGIT as an epigenetically regulated immunomodulator affecting immunotherapeutic sensitivity of gastric cancer. Cancer Biomark..

[31] Yang Z-Z (2020). TIGIT expression is associated with t-cell suppression and exhaustion and predicts clinical outcome and anti-PD-1 response in follicular lymphoma. Clin. Cancer Res..

[32] Shaffer T, Natarajan A, Gambhir SS (2021). PET imaging of TIGIT expression on tumor-infiltrating lymphocytes. Clin. Cancer Res..

[33] Zhang J-A (2020). Development of an immune-related gene signature for prognosis in melanoma. Front. Oncol..

[34] Farrow NE (2021). Characterization of sentinel lymph node immune signatures and implications for risk stratification for adjuvant therapy in melanoma. Ann. Surg. Oncol..

[35] Inozume T (2016). Melanoma cells control antimelanoma CTL responses via interaction between TIGIT and CD155 in the effector phase. J. Invest. Dermatol..

[36] Joller N (2011). Cutting edge: TIGIT has T cell-intrinsic inhibitory functions. J. Immunol..

[37] Liu S (2013). Recruitment of Grb2 and SHIP1 by the ITT-like motif of TIGIT suppresses granule polarization and cytotoxicity of NK cells. Cell Death Differ..

[38] Joller N (2014). Treg cells expressing the coinhibitory molecule TIGIT selectively inhibit proinflammatory Th1 and Th17 cell responses. Immunity.

[39] Fourcade J (2018). CD226 opposes TIGIT to disrupt Tregs in melanoma. JCI Insight.

[40] Lozano E, Dominguez-Villar M, Kuchroo V, Hafler DA (2012). The TIGIT/CD226 axis regulates human T cell function. J. Immunol..

[41] Du X (2018). CD226 regulates natural killer cell antitumor responses via phosphorylation-mediated inactivation of transcription factor FOXO1. Proc. Natl. Acad. Sci. U. S. A..

[42] Rishiq A, Bsoul R, Pick O, Mandelboim O (2023). Studying TIGIT activity against tumors through the generation of knockout mice. Oncoimmunology.

[43] Li J (2022). Single-cell characterization of the cellular landscape of acral melanoma identifies novel targets for immunotherapy. Clin. Cancer Res..

