# Supplementary material for: Single-cell RNA sequencing unveils tumor heterogeneity and immune microenvironment between subungual and plantar melanoma
Source: Sci Rep. 2024 Mar 25;14:7039. doi: 10.1038/s41598-024-57640-8 (PMC10963724; doi:10.1038/s41598-024-57640-8)
Supplement: Supplementary file 1 — Supplementary Legends. [file 41598_2024_57640_MOESM1_ESM.docx]

**Legends for supplementary tables**

**Supplementary Table1**. Total number of cells in the four samples.

**Supplementary Table2**. Number and percentage of cells of different cell types in the four samples.

**Supplementary Table3**. Results of cellchat between fibroblast subtypes and melanocyte subtypes

**Supplementary Table4**. Summary of genes in different modules in the fibroblast subtypes pseudo-time analysis.

**Supplementary Table5**. Results of cellchat between T/NK cell subtypes and melanocyte subtypes.

**Supplementary Table6**. Summary of genes in different modules in the T/NK cell subtypes pseudo-time analysis.
