# Supplementary material for: Single-cell RNA sequencing unveils tumor heterogeneity and immune microenvironment between subungual and plantar melanoma
Source: Sci Rep. 2024 Mar 25;14:7039. doi: 10.1038/s41598-024-57640-8 (PMC10963724; doi:10.1038/s41598-024-57640-8)
Supplement: Supplementary file 2 — Supplementary Information. [file 41598_2024_57640_MOESM2_ESM.docx]

**1. Single-cell RNA-seq data preprocessing**

The Cell Ranger software pipeline (version 5.0.0) provided by 10×Genomics was used to demultiplex cellular barcodes, map reads to the genome and transcriptome using the STAR aligner, and down-sample reads as required to generate normalized aggregate data across samples, producing a matrix of gene counts versus cells. We processed the unique molecular identifier (UMI) count matrix using the R package Seurat^[1]^ (version 3.1.1). To remove low quality cells and likely multiplet captures, which is a major concern in microdroplet-based experiments, we applied a criteria to filter out cells with gene numbers less than 200, UMI less than 1000 and log10GenesPerUMI less than 0.7. We further discarded low-quality cells where >10% of the counts belonged to mitochondrial genes and >5% of the counts belonged to hemoglobin genes. Additionally, we applied DoubletFinder package^[2]^ (version 2.0.2) to identify potential doublet. After applying these QC criteria, 24,789 single cells were included in downstream analyses. Library size normalization was performed with NormalizeData function in Seurat^[1]^ to obtain the normalized count. Specifically, the global-scaling normalization method “LogNormalize” normalized the gene expression measurements for each cell by the total expression, multiplied by a scaling factor (10,000 by default), and the results were logtransformed.

Top variable genes across single cells were identified using the method described in Macosko et al^[3]^. The most variable genes were selected using FindVariableGenes function(mean.function = FastExpMean, dispersion.function = FastLogVMR) in Seurat^[1]^. To remove the batch effects in single-cell RNA-sequencing data, the mutual nearest neighbors(MNN) presented by Haghverdi et al was performed with the R package batchelor^[4]^. Graph-based clustering was performed to cluster cells according to their gene expression profile using the FindClusters function in Seurat^[1]^. Cells were visualized using a 2-dimensional Uniform Manifold Approximation and Projection (UMAP) algorithm with the RunUMAP function in Seurat^[1]^. We used the FindAllMarkers function(test.use = presto) in Seurat^[1]^ to identify marker genes of each cluster. For a given cluster, FindAllMarkers identified positive markers compared with all other cells. Then, we used the R package SingleR^[5]^, a novel computational method for unbiased cell type recognition of scRNA-seq, with the reference transcriptomic datasets ‘Human Primary Cell Atlas’ ^[6]^ to infer the cell of origin of each of the single cells independently and identify cell types.

Differentially expressed genes(DEGs) were identified using the FindMarkers function(test.use = presto) in Seurat[1]. P value < 0.05 and |log2foldchange| > 0.58 was set as the threshold for significantly differential expression. GO enrichment and KEGG pathway enrichment analysis of DEGs were respectively performed using R based on the hypergeometric distribution.

The sequencing and bioinformatics analysis were performed by OE Biotech Co., Ltd. (Shanghai, China).

[1]Butler A, Hoffman P, Smibert P, et al. Integrating single-cell transcriptomic data across different conditions, technologies, and species[J]. Nature biotechnology, 2018, 36(5): 411-420.

[2]Mcginnis C S , Murrow L M , Gartner Z J . DoubletFinder: Doublet detection in single-cell RNA sequencing data using artificial nearest neighbors. 2018.

[3]Macosko E Z, Basu A, Satija R, et al. Highly parallel genome-wide expression profiling of individual cells using nanoliter droplets[J]. Cell, 2015, 161(5): 1202-1214.

[4]Haghverdi L, Lun A T L, Morgan M D, et al. Batch effects in single-cell RNA-sequencing data are corrected by matching mutual nearest neighbors[J]. Nature biotechnology, 2018, 36(5): 421-427.

[5]Aran D, Looney A P, Liu L, et al. Reference-based analysis of lung single-cell sequencing reveals a transitional profibrotic macrophage[J]. Nature immunology, 2019, 20(2): 163-172.

[6]Mabbott N A, Baillie J K, Brown H, et al. An expression atlas of human primary cells: inference of gene function from coexpression networks[J]. BMC genomics, 2013, 14(1): 632.

**2. Pseudotime analysis**

We determined the developmental pseudotime with the Monocle2 package^[1]^. The raw count was first converted from Seurat object into CellDataSet object with the importCDS function in Monocle. We used the differentialGeneTest function of the Monocle2 package to select ordering genes (qval < 0.01) which were likely to be informative in the ordering of cells along the pseudotime trajectory. The dimensional reduction clustering analysis was performed with the reduceDimension function, followed by trajectory inference with the orderCells function using default parameters. Gene expression was plotted with the plot_genes_in_pseudotime function to track changes over pseudo-time.

[1] Trapnell C, Cacchiarelli D, Grimsby J, et al. The dynamics and regulators of cell fate decisions are revealed by pseudotemporal ordering of single cells[J]. Nature biotechnology, 2014, 32(4): 381.

**3. SCENIC analysis**

The SCENIC analysis was run using the motifs database for RcisTarget and GRNboost (SCENIC^[1]^ version 1.1.2.2, which corresponds to RcisTarget 1.2.1 and AUCell 1.4.1) with the default parameters. In detail, we identified transcription factor (TF) binding motifs over-represented on a gene list with RcisTarget package. The activity of each group of regulons in each cell was scored by AUCell package.

To evaluate the cell type specificity of each predicted regulon, we calculated the regulon specificity score (RSS) which was based on the Jensen-Shannon divergence (JSD), a measure of the similarity between two probability distributions. Specifically, we calculated the JSD (Jensen-Shannon divergence) between each vector of binary regulon activity overlaps with the assignment of cells to a specific cell type^[2]^. The connection specificity index (CSI) for all regulons was calculated with the scFunctions (<https://github.com/FloWuenne/scFunctions/>) package.

[1] Aibar S, González-Blas C B, Moerman T, et al. SCENIC: single-cell regulatory network inference and clustering[J]. Nature methods, 2017, 14(11): 1083-1086.

[2] Suo S , Zhu Q , Saadatpour A , et al. Revealing the Critical Regulators of Cell Identity in the Mouse Cell Atlas[J]. Cell Reports, 2018, 25(6):1436-1445.e3.

**4. Gene set variation analysis (GSVA)**

To perform the Gene Set Variation Analysis, the GSEABase package (version 1.44.0) was used to load the gene set file which was downloaded and processed from KEGG database (<https://www.kegg.jp/>). To assign pathway activity estimates to individual cells, we applied GSVA^[1]^ using standard settings, as implemented in the GSVA package (version 1.30.0). The differences in pathway activities scored per cell were calculated with LIMMA package (version 3.38.3).

[1] Hänzelmann S, Castelo R, Guinney J. GSVA: gene set variation analysis for microarray and RNA-seq data[J]. BMC bioinformatics, 2013, 14(1): 7.

**5.****CNV analysis**

Initial CNVs for each region were estimated by inferCNV^[1]^ R package. The CNV of total cell types was calculated by expression level from single-cell sequencing data for each cell with -- cutoff 0.01.

Genes were sorted based on their chromosomal location and a moving average of gene expression was calculated using a window size of 101 genes. The expression was then centered to zero by subtracting the mean. The melanocytes were selected as malignant cells, leaving all remaining cells as the normal cells. The de-noising was carried out to generate the final CNV profiles.

1. Puram S V, Tirosh I, Parikh A S, et al. Single-cell transcriptomic analysis of primary and metastatic tumor ecosystems in head and neck cancer[J]. Cell, 2017, 171(7): 1611-1624. e24.
2. **CellChat**

Cell-to-cell ligand-receptor interaction analysis was performed using the CellChat^[1]^ (v 1.1.3) R package. Firstly, the normalized expression matrix was imported and cellchat object was created through createCellChat function; using default parameters through identify Over Expressed Genes, identify Over Expressed Interactions, and project Data functions for the preprocessing operations; calculate potential ligand-receptor interactions using the computeCommunProb, filterCommunication (min.cells = 10), and computeCommunProbPathway functions; and finally aggregate intercellular communication networks using the aggregateNet function.

[1] Jin S, Guerrero-Juarez CF, Zhang L, et al. Inference and analysis of cell-cell communication using CellChat. Nat Commun. 2021;12(1):1088. Published 2021 Feb 17. doi:10.1038/s41467-021-21246-9
